# Methods and Annotated Data Sets Used to Predict the Gender and Age of Twitter Users: Scoping Review

**DOI:** 10.2196/47923

**Published:** 2024-03-15

**Authors:** Karen O'Connor, Su Golder, Davy Weissenbacher, Ari Z Klein, Arjun Magge, Graciela Gonzalez-Hernandez

**Affiliations:** 1 Department of Biostatistics, Epidemiology and Informatics Perelman School of Medicine University of Pennsylvania Philadelphia, PA United States; 2 Department of Health Sciences University of York York United Kingdom; 3 Department of Computational Biomedicine Cedars-Sinai Medical Center Los Angeles, CA United States

**Keywords:** social media, demographics, Twitter, age, gender, prediction, real-world data, neural network, machine learning, gender prediction, age prediction

## Abstract

**Background:**

Patient health data collected from a variety of nontraditional resources, commonly referred to as *real-world data*, can be a key information source for health and social science research. Social media platforms, such as Twitter (Twitter, Inc), offer vast amounts of real-world data. An important aspect of incorporating social media data in scientific research is identifying the demographic characteristics of the users who posted those data. Age and gender are considered key demographics for assessing the representativeness of the sample and enable researchers to study subgroups and disparities effectively. However, deciphering the age and gender of social media users poses challenges.

**Objective:**

This scoping review aims to summarize the existing literature on the prediction of the age and gender of Twitter users and provide an overview of the methods used.

**Methods:**

We searched 15 electronic databases and carried out reference checking to identify relevant studies that met our inclusion criteria: studies that predicted the age or gender of Twitter users using computational methods. The screening process was performed independently by 2 researchers to ensure the accuracy and reliability of the included studies.

**Results:**

Of the initial 684 studies retrieved, 74 (10.8%) studies met our inclusion criteria. Among these 74 studies, 42 (57%) focused on predicting gender, 8 (11%) focused on predicting age, and 24 (32%) predicted a combination of both age and gender. Gender prediction was predominantly approached as a binary classification task, with the reported performance of the methods ranging from 0.58 to 0.96 *F*_1_-score or 0.51 to 0.97 accuracy. Age prediction approaches varied in terms of classification groups, with a higher range of reported performance, ranging from 0.31 to 0.94 *F*_1_-score or 0.43 to 0.86 accuracy. The heterogeneous nature of the studies and the reporting of dissimilar performance metrics made it challenging to quantitatively synthesize results and draw definitive conclusions.

**Conclusions:**

Our review found that although automated methods for predicting the age and gender of Twitter users have evolved to incorporate techniques such as deep neural networks, a significant proportion of the attempts rely on traditional machine learning methods, suggesting that there is potential to improve the performance of these tasks by using more advanced methods. Gender prediction has generally achieved a higher reported performance than age prediction. However, the lack of standardized reporting of performance metrics or standard annotated corpora to evaluate the methods used hinders any meaningful comparison of the approaches. Potential biases stemming from the collection and labeling of data used in the studies was identified as a problem, emphasizing the need for careful consideration and mitigation of biases in future studies. This scoping review provides valuable insights into the methods used for predicting the age and gender of Twitter users, along with the challenges and considerations associated with these methods.

## Introduction

### Background

Real-world data are data regarding patients’ health collected outside randomized controlled trials from a variety of nontraditional resources such as electronic health records, medical claims data, or data generated by patients themselves such as social media data that may be used to support study design to develop real-world evidence [1]. Real-world data from social media have been increasingly recognized as a valuable resource for gaining knowledge about and insight into a variety of health-related research topics, including disease surveillance [2,3], pharmacovigilance [4,5], and mental health [6,7]. They can also be used for the identification of cohorts for potential recruitment into traditional studies [8,9]. In short, social media can readily provide abundant personal health information in real time.

The use of data from social media platforms, particularly Twitter (Twitter, Inc), for health-related research is subject to some inherent limitations in that demographic information (with the exception of location, which is available when the user has enabled the location feature) is not explicitly available through the application programming interface (API) [10]. Demographic traits, including age, gender, race or ethnicity, location, education, and income, hold significant value in health research. Few studies based on Twitter data incorporated an assessment of Twitter user demographics into their analysis [11]. Understanding the demographic traits of Twitter users provides significant value when using the data in health research. It not only facilitates sample representativeness, which is crucial for generalizing research findings and ensuring that the conclusions drawn from Twitter data can be extrapolated to broader populations [12], but also enables subgroup analysis. It allows for the comparison of health-related behaviors, attitudes, and outcomes across different groups and enables targeted interventions and tailored health care strategies [13,14]. Moreover, demographic information is actionable and can assist in designing public health interventions and policies for specific populations based on their needs and concerns as expressed on social media.

Predicting demographic traits is complex and challenging. A user’s profile does not necessarily include such information, and researchers have used other features available in the data, such as names, content of the tweets, or the individual’s network to make predictions. A 2018 systematic review assessed the use of social media to predict demographic traits, finding successful implementation for 14 traits, including gender and age [15,16]. Although the review provided a broad overview of the state of demographic prediction using social media, the details of the machine learning (ML) methods used were not reviewed. A recent review provided insights into the methods used for predicting the race and ethnicity of Twitter users [17].

### Objectives

In this study, our objective was to present a scoping review of automated methods used for predicting the age and gender of Twitter users to provide an overview of the techniques published since 2017. We focused our review on studies that used Twitter, as it is the most commonly used social media platform for this research [15]. Twitter is an attractive platform to use in research, as the terms of use for this platform are well understood by both users and researchers, it includes an API, and the data on it are abundant for health-related research [18].

Although other demographic traits such as location, education, and income can provide valuable insights, the age and gender of Twitter users present distinct advantages and considerations for health research. Given the differences in disease presentation by gender, such as with acute coronary syndrome [19], and by age, such as with COVID-19 [20], identifying the age and gender of the users included in studies using Twitter data may elicit insights into disease prevalence, patterns, and variations across different subgroups in disease presentation or treatment response [21,22]. Age and gender also play crucial roles in shaping health behaviors and attitudes. For example, studying age and gender differences in smoking habits [23], physical activity levels [24], and adherence to medical treatments [25,26] can provide insights into effective interventions and health promotion campaigns for specific groups. Although Twitter users are generally representative of the population, there is a certain degree of skew in their demographics: there is an overrepresentation of individuals aged <30 years, whereas individuals aged >65 years are underrepresented when compared with the overall demographics of the US population [27,28]. Therefore, it is important to include the age and gender of Twitter users in a study to enable the accurate reporting of findings, making them specific to certain subgroups, or to make any necessary adjustments to account for potential biases that may arise from these demographic differences.

Although studies aimed at predicting Twitter users’ gender began as early as 2011 [29-33] and efforts aimed at detecting the age of Twitter users have been made since 2013 [34-36], it is only since 2017 that the language processing community shifted its methods away from handcrafted rules and represented text documents with dense vectors to train deep neural networks (DNNs) [37,38], resulting in a noticeable increase in performance for many applications. We sought to examine whether these increases in performance were evident in the methods used for the prediction of the age and gender of Twitter users.

## Methods

### Overview

We report this review following the PRISMA-ScR (Preferred Reporting Items for Systematic Reviews and Meta-Analyses Extension for Scoping Reviews) [39] methodology. The completed PRISMA-ScR checklist is available in Table S1 in Multimedia Appendix 1. We searched several databases to identify studies on the prediction of Twitter users’ age or gender or both. Our database search strategy combines 3 facets: facet 1 includes terms related to Twitter, facet 2 consists of terms for age or gender, and facet 3 consists of terms for methods of prediction such as ML. The search strategy was translated as appropriate for each database. The detailed search strategy is available in Multimedia Appendix 2. The ML term facet was expanded using terms from related reviews by Hinds and Joinson [15] and Umar et al [40]. The search criteria were limited to peer-reviewed journals, conference proceedings, books, and theses.

The following databases were searched with a publication date range of 2017 or later (Textbox 1).

**List of databases searched with the total number of combined facet results.**

- ACL (Association for Computational Linguistics) Anthology: 5080, of which the first 50 records were screened
- ACM (Association for Computing Machinery) Digital Library: 23
- Cumulative Index to Nursing & Allied Health (CINAHL): 57
- Embase: 262
- Google Scholar: 767,000, of which the first 50 records were screened
- IEEE (Institute of Electrical and Electronics Engineers) Xplore: 23
- Library and Information Science Abstracts: 31
- Library, Information Science and Technology Abstracts: 48
- Proquest Dissertations and Theses—United Kingdom and Ireland: 58
- Ovid MEDLINE: 183
- PsycINFO: 104
- Science Citation Index, Social Science Citation Index, Conference Proceedings Citation Index—Science, and Conference Proceedings Citation Index—Social Science and Humanities: 131
- Zetoc: 61

Citations were exported to a shared EndNote (Clarivate) library for deduplication. Using the Population, Intervention, Comparison, Outcomes, and Study Design (PICOS) [41] framework, we developed a list of inclusion and exclusion criteria (refer to the *Inclusion and Exclusion Criteria* section), and 2 screeners from the research team screened the results independently, with disputes discussed after screening and a consensus decision reached. In addition, given that search engines and unmanageable data sources are recommended to be included as secondary data sources [42-44], the first 50 records from both ACL (Association for Computational Linguistics) Anthology and Google Scholar were screened using the aforementioned methods. We set a limit on the number of results screened, as the relevance of the results is ranked by the search engines, with the most relevant results listed first [45-48].

### Inclusion and Exclusion Criteria

We framed our research question using the PICOS framework. Table 1 outlines our specific inclusion and exclusion criteria. As explained in the *Introduction* section, we restricted the date of our search to include only publications from 2017 and beyond. No language restrictions were applied to the inclusion criteria; however, financial and logistical restraints allowed us to include only studies written in English, Spanish, Chinese, or French.

**Table 1:** Inclusion and exclusion criteria, developed per the Population, Intervention, Comparison, Outcomes, and Study Design framework, for the scoping review.

Facet	Inclusion criteria	Exclusion criteria
Population	Any Twitter (Twitter, Inc) data on Twitter users, such as posts, profile details, photos, or avatars	Studies evaluating prediction from data on other social media platforms, such as Facebook (Meta Platforms, Inc) or Instagram (Meta Platforms, Inc)
Intervention	Methods for predicting the gender or age of Twitter users; articles that used machine learning, natural language processing, human in the loop, or other computationally assisted methods to predict the gender or age of the users	Studies that contained no computation methods
Comparator	Any or none; we included any studies irrespective of whether they had a comparator and, if they did have a comparator, irrespective of what that was	N/A^a^
Outcome	Gender or age prediction	Any other demographic trait prediction
Study design	Any type of peer-reviewed study reporting on the methods used to predict gender or age; such information must be the primary focus of the study or reported in enough detail to be reproducible	Discussion papers, commentaries, and letters
Date	2017 or later	Before 2017
Language	All	None

^a^N/A: not applicable.

### Data Extraction

From each included paper, we extracted the following data: the year of publication, publication type (journal, conference paper, book chapter, or thesis), demographic predicted (gender, age, or both), language of tweets, size of the data set, collection method for the data set, details of prediction models, features used in the models (posts, profile, and images), performance of the models, name of any software used for prediction, measures used to assess the methods and results of any evaluation, and the availability of data or code. The included papers were distributed among the authors for data extraction. The extracted data were validated by another author (KO).

## Results

### Overview

Our database searches resulted in 981 studies, which were retrieved and entered into an EndNote library, where duplicates were removed, leaving 684 (69.7%) studies for sifting.

**Figure 1:**
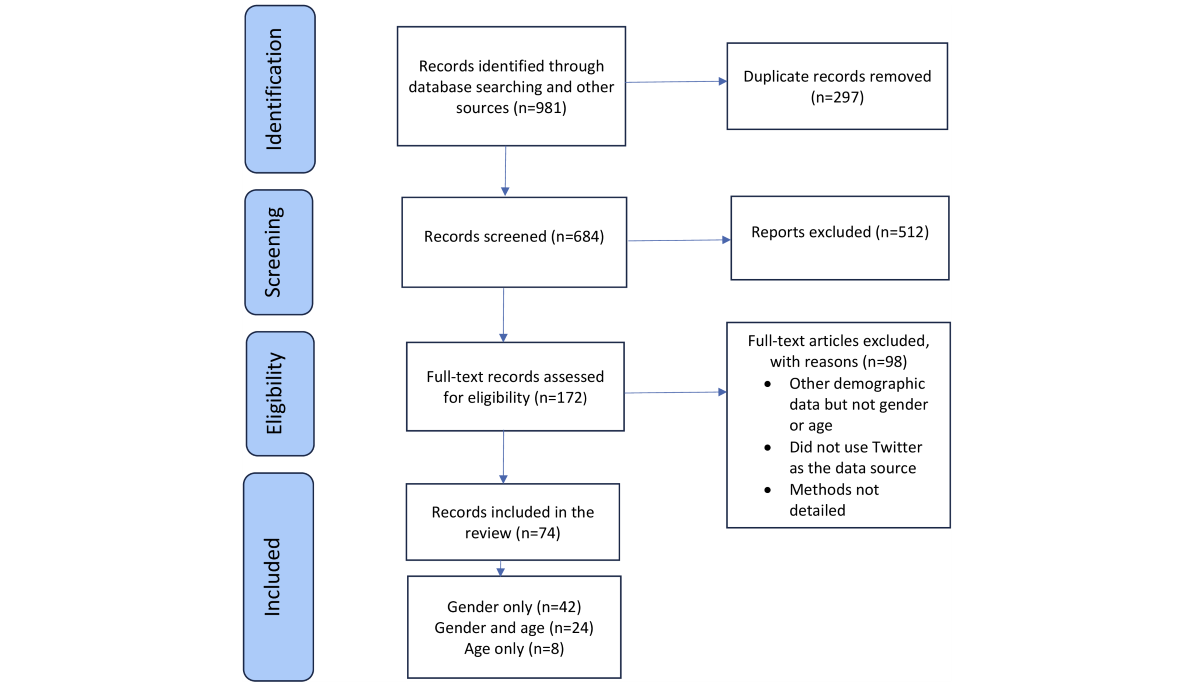
PRISMA (Preferred Reporting Items for Systematic Reviews and Meta-Analyses) flow diagram of the included studies.

After the abstract review, 172 (25.1%) of 684 studies were deemed potentially relevant by either one of the independent sifters (SG and KO). The full texts of these studies were screened independently, and disagreements were discussed, resulting in the inclusion of 74 (43%) studies [49-122] and exclusion of 98 (57%) studies (Figure 1).

### Characteristics of the Included Studies

Among the 74 included studies (Multimedia Appendices 3 [49-52,54-63,65,67-72,74-89,91-93,96-99,101-122] and 4 [51,53,55,56,59,60,63-67,70,73,74,77,80,83,84,87,90,94, 95,99-101,108,110,112,116,118-120]), the majority (n=42, 57%) focused on predicting only the gender of the individual, 24 (32%) explored predicting both gender and age, and 8 (11%) focused solely on predicting age. Most of the studies were published in conference proceedings (44/74, 59%), followed by journal articles (28/74, 38%), theses (2/74, 3%), and a book chapter (1/74, 1%).

In 42 (57%) of the 74 studies, developing methods to predict Twitter users’ age or gender or both was the primary purpose. In the remaining studies (32/74, 43%), the identification of the demographic characteristics of Twitter users was secondary. Within this last group, 9 (28%) studies developed ad-hoc methods to determine age, gender, or both, whereas the others used open-source models (13/32, 41%) or off-the-shelf software (10/32, 31%).

### Studies Developing Ad-Hoc Methods for Gender and Age Prediction

#### Gender

##### Overview

Of the 74 studies, 44 (59%) developed ad-hoc methods to predict the Twitter users’ gender. Of these 44 studies, 32 (73%) predicted the users’ gender alone [49,50,52,54,57,58,68,69, 71,72,75,76,79,81,82,85,86,89,92,93,96,102,104-107,111,113, 115,117,121,122], and 12 (27%) predicted gender along with age [51,55,65,70,80,83,87,101,108,110,112,116].

Most studies that developed ad-hoc methods (41/44, 93%) approached the problem of gender prediction as a binary classification task, predicting whether the label male or female applies to each user account, whereas 4% (3/44) of studies [93,112,119] added the classification of organization or brand.

We found that approaches to predict gender included tweets written in multiple languages, including English [52,82,83,92,93,115,117], German [76], Slovenian [106], Italian [49], Japanese [89], Arabic and Egyptian [57,58,79], French, Dutch, Portuguese, and Spanish, and a multilingual study assessed tweets written in 28 languages and dialects [112].

##### Data Sets

For the training and validation of the ad-hoc approaches for gender detection, some studies (19/44, 43%) used previously created annotated corpora, whereas others (27/44, 61%) collected data directly from Twitter. Among the 19 studies that used previously annotated data sets, 9 (47%) [55,57,58,68,70,86,87,96,121] used corpora from the PAN-Conference and Labs of the Evaluation Forum (CLEF; PAN-CLEF) author profiling tasks [123-129], whereas 10 (53%) studies [72,75,83,85,93,104,105,115,117,122] relied on data sets from other studies [113,130-136].

In the 27 (61%) studies that collected data directly from Twitter, different components of Twitter accounts were used. These components were used either for manually or semiautomatically validating the gender of a user or for computing features describing the user to train a classifier (Multimedia Appendix 5 [49-122]). Despite data limitations from the Twitter API, it was the main source of data collection, with 22 (24%) studies [49-52,54,69,71,76,79,81,89,92,101,102,106-108,110,111,116,117,121] collecting data either as a random sample from the Twitter Streaming API or based on keywords or geographic location from the Twitter Search API. Of the 5 studies not using the Twitter API, 1 (20%) [82] collected data using a scraping tool, 3 (60%) [80,112,113] used a random sample from a collection of 10% of tweets from 2014 to 2017 or the Twitter archive, and 1 (20%) did not specify its data source [65].

The 24 studies that created a labeled data set (Multimedia Appendix 6 [49,51-54,63,64,66,69,71,73,76,77,80,82,89,90,92, 106-108,110,112,113,116-118,120]) to train and test or to validate the performance of the system determined the gender of the users using multiple components of their Twitter accounts (Multimedia Appendix 5). A total of 11 (46%) studies labeled the data through manual annotation, where the annotators determined the gender using profile pictures [52,54], user names [71], profiles [89], or a combination of these [76,82,92,106,108,110,116]. There were 11 (46%) studies that automatically or semiautomatically labeled their data sets via the detection of self-reports or gender-identifying terms (eg, mother, son, and uncle) [69,80,108,110,112,117], the user’s name [49,107,113], or declarations on other linked social media [116,117]. A total of 3 (13%) studies created their labeled data sets by using the accounts of famous social media influencers [65] or using an unspecified collection of users whose gender is known [51,79]. Of the 24 studies, only 8 (33%) reported data availability. Of the 8 studies, 6 (75%) stated availability *by request*, and 2 (25%) had working links to the whole corpus (Multimedia Appendix 6).

##### Nonpersonal Accounts

A Twitter account may not be authored by or represent a single person. There are organization or company accounts as well as *bot* accounts. A bot is an automatic or semiautomatic user account. Some bot accounts identify themselves as such and may be used to automatically amplify news or tweets related to a certain topic. Others may emulate human accounts and be used with a more malicious intent to sow discord, manipulate public opinion, or spread misinformation. There were 9 (12%) of the 74 included studies [49,76,92,93,96,103,104,106,112] that removed nonpersonal (organization) accounts when they manually annotated their collections. Some studies (11/74, 15%) implemented heuristics to explicitly detect and remove nonpersonal accounts [49,50,59,71,81,107,113,122], bot accounts [98], or both [79,137]. Others (39/74, 53%) used previously annotated data sets consisting of only personal accounts, labeled and removed nonpersonal accounts, or collected their data sets based on self-reports of age and gender or other identifiable personal information. The remaining (15/74, 20%) studies provided no details on how or whether these accounts were removed (Multimedia Appendix 5).

##### Features and Models

The reviewed studies used data labeled with the user’s gender to build and evaluate classification models based on features describing the tweets (such as n-grams, word embeddings, hashtags, and URLs) [57,58,65,68-71,75,79,82,86,87,92, 96,104,109,113,121], features derived from the users’ profile metadata (such as user names, bio, followers, and users followed) [49,51,52,72,80,85,112,115,122], features derived from a combination of their profile metadata and tweets [52,54,76,83,93,107,108,110,117] or images [52,80,108,112, 116]. Of the 74 studies, 1 (3%) study from Japan included the user’s geographic information under the assumption that, culturally, a person of a certain demographic is more likely to frequent specific places [89].

Among the systems that used handcrafted features (25/44, 57%), most (13/25, 52%) achieved their best results using a support vector machine (SVM) [49,54,65,72,82,85,86,104-106, 113,116,138], whereas others (12/25, 48%) used logistic regression [87,107,110], naive Bayes [51,92], random forests [80], bag of trees [70], extreme gradient boosting [89], or ensemble approaches [76,79,107,122] (details are provided in Table 2). Other systems used deep learning methods (15/44, 34%) such as DNNs, convolutional neural networks, feed forward neural networks or recurrent neural networks [55,68,71,75,93,115,121], bidirectional long-term short-term memory [58], gated recurrent units [57], graph recursive neural networks [83], and multimodal deep learning networks [108,112].

One of the studies created a meta-classifier ensemble classifying users based on the predictions of multiple individual classifiers [117], including SVM, bidirectional encoder representations from transformers, and 2 existing models [112,139]. Another study created a DNN for learning with label proportion, a semisupervised approach [52]. The results of the best-performing deep learning model as reported in each study are presented in Table 3. Studies that used lexical matching (4/44, 9%) of the user’s name to a curated name dictionary [50,81,101,102] to determine gender reported no validation or performance metrics.

**Table 2:** Top reported system performance for studies predicting the gender of Twitter users using traditional machine learning (ML) methods. Result metrics are reflected in this table as reported in the original publications and are not necessarily comparable to each other.

Study	Language	ML method	Reported performance	
			*F*_1_-score	Accuracy
Cesare et al [122], 2017	English	Ensemble: lexical match and SVM^a^ and DT^b^	0.84	0.83
Jurgens et al [80], 2017	English	RF^c^ ensemble	0.78	0.80
Ljubešić et al [85], 2017	Portuguese, French, Dutch, Spanish, German, and Italian	SVM	N/A^d^	0.61-0.69
Markov et al [87], 2016	English, Spanish, Dutch, and Italian	LogR^e^	N/A	0.57-0.77
Mukherjee and Bala [92], 2016	English	NB^f^	0.75	0.71
Verhoeven et al [106], 2017	Slovenian	SVM	0.93	0.93
Volkova [110], 2015	English and Spanish	LogR	N/A	0.82
Xiang et al [116], 2017	English	SVM and PME^g^	N/A	0.76
Cheng et al [65], 2018	English, Filipino, and Taglish	SVC^h^ with lasso	0.84	0.84
Emmery et al [69], 2017	English	fastText	N/A	0.76
Giannakopoulos et al [72], 2018	N/A	SVM PNN^i^	N/A	0.87
Khandelwal et al [82], 2018	Code-mixed Hindi-English	SVM	N/A	0.9
Miura et al [89], 2018	Japanese	XGBoost^j^	N/A	0.89
van der Goot et al [104], 2018	English, Dutch, French, Portuguese, and Spanish	SVM	N/A	0.66-0.72
Alessandra et al [49], 2019	Italian	Ensemble: lexical match and SVM	N/A	0.75
Hirt et al [76], 2019	German	Ensemble: binary classifiers	0.81	N/A
Hussein et al [79], 2019	Dialect Egyptian Arabic	Ensemble: RF and LinR^k^	NA	0.77-0.88
Vicente et al [107], 2018	English and Portuguese	Ensemble: Face++, LinR, and SVM	N/A	0.93-0.97
Arafat et al [51], 2020	Indonesian	Multinomial NB	N/A	0.75
Baxevanakis et al [54], 2020	Greek	SVM	N/A	0.7
Garcia-Guzman et al [70], 2020	English	Bag of trees	0.64	0.64
López-Monroy et al [86], 2020	English and Spanish	Bag of trees	0.64	0.64
Pizarro [96], 2020	English and Spanish	SVM	0.82-0.84	N/A
Vashisth and Meehan [105], 2020	English	LogR	N/A	0.57
Wong et al [113], 2020	English	SVM	0.58-0.62	0.60

^a^SVM: support vector machine.

^b^DT: decision tree.

^c^RF: random forest.

^d^N/A: not applicable.

^e^LogR: logistic regression.

^f^NB: naive Bayes.

^g^PME: projection matrix extraction.

^h^SVC: support vector classifier.

^i^PNN: probabilistic neural network.

^j^XGBoost: extreme gradient boosting.

^k^LinR: linear regression.

**Table 3:** Top reported system performance for studies predicting the gender of Twitter users using deep learning machine learning (ML) methods. Result metrics are reflected in this table as reported in the original publications and are not comparable to each other.

Study	Language	ML method	Reported performance	
			*F*_1_-score	Accuracy
Ardehaly and Culotta [52], 2017	English	Deep LLP^a^	0.96	N/A^b^
Geng et al [71], 2017	English	Ensemble: LDA^c^ and CNN^d^	N/A	0.87
Kim et al [83], 2017	English	GRNN^e^	N/A	0.68
Vijayaraghavan et al [108], 2017	English	DMT^f^	0.89	N/A
Wang et al [111], 2017	N/A	CNN	0.91	0.9
Bayot and Goncalves [55], 2017	English and Spanish	CNN	N/A	0.59-0.72
Bsir and Zrigui [57], 2018	Arabic	GRU^g^	N/A	0.79
Wood-Doughty et al [115], 2018	English	RNN^h^	0.84	0.84
Bsir and Zrigui [58], 2019	Arabic	BILSTM^i^ with attention	N/A	0.82
Hashempour [75], 2019	Portuguese, French, Dutch, Spanish, German, and Italian	FFNN^j^	N/A	0.84-0.86
Wang et al [112], 2019	Multilingual	mmDNN^k^	0.92	N/A
ElSayed and Farouk [68], 2020	Egyptian and Arabic dialects	Multichannel CNN-biGRU^l^	N/A	0.84-0.91
Imuede et al [93], 2020	English	DNN^m^	N/A	0.68
Zhao et al [121], 2020	English	CNN	0.80	N/A
Yang et al [117], 2021	English	Ensemble: M3^n^ and SVM^o^	0.95	0.94

^a^LLP: learning with label proportions.

^b^N/A: not applicable.

^c^LDA: latent Dirichlet allocation.

^d^CNN: convolutional neural network.

^e^GRNN: graph recurrent neural network.

^f^DMT: deep multimodal multitask.

^g^GRU: gated recurrent network.

^h^RNN: recurrent neural network.

^i^BILSTM: bidirectional long-term short-term memory.

^j^FFNN: feed forward neural network.

^k^mmDNN: multimodal deep neural network.

^l^biGRU: bidirectional gated recurrent unit.

^m^DNN: deep neural network.

^n^M3: multimodal, multilingual, and multi-attribute system.

^o^SVM: support vector machine.

##### Performance

Performance results from the traditional ML methods cannot be directly compared against the deep learning methods used, as they were evaluated against different gold-standard corpora, and they used nonstandardized reporting metrics. However, looking at the overall results in terms of *F*_1_-score, the results of the studies using deep learning had a relatively narrower range of reported performance (0.84-0.96), with a higher minimum of 0.84 and higher maximum of 0.96, compared with the reported performance range for traditional ML methods, which spans from 0.64 to 0.93.

#### Age

##### Overview

We found 19 studies that developed ad-hoc methods to predict the Twitter user’s age, among which 7 (37%) predicted age exclusively [53,64,66,73,90,94,95]. All but 1 (5%) of the studies [80] approached the detection of Twitter users’ age as an automatic classification of predefined age groups. The number of age groups varied across the studies (Table 3), with the ages categorized into 2 [53,73,83,110,116], 3 [51,66,90, 94,95,101,108], 4 [70,112], or more [55,64,65,87] groups. The range of ages within the groups also varied across the studies; for example, across the 5 studies that took a binary classification approach, Guimaraes et al [73] used 13 to 19 years and ≥20 years as the 2 age groups, Volkova et al [110] and Kim et al [83] used 18 to 23 years or ≥25 years, Xiang et al [116] used ≤30 years or >30 years, and Ardehaly and Culotta [53] used <25 years and ≥25 years.

Except for 2 (11%) studies that did not report the language of the tweets used [51,73], all studies used English language tweets. A total of 8 (42%) studies extended their systems to include additional languages, including Spanish [55,64,87,110], Dutch [87,94,95], Filipino [65], and multiple languages [112].

##### Data Sets

Most studies (9/19, 47%) that developed new algorithms prepared new data sets to evaluate them with data retrieved directly using Twitter’s API [51,53,66,73,90,108] or used other sources of data for this purpose [64,80,112] (Multimedia Appendix 4). Several studies used data sets made available by other studies to train or evaluate their algorithms: among the 19 studies, 2 (11%) studies [94,95] combined data sets from Sloan et al [34], Nguyen et al [36], and Morgan-Lopez et al [90]; Kim et al [83] used the data set from Volkova et al [140]; and 3 (15%) studies [55,70,87] used data sets that were created for the PAN-CLEF author profiling shared tasks [124-126]. The studies that prepared new data sets (Multimedia Appendix 6) labeled users’ age groups by automatically or semiautomatically searching (1) for tweets that self-reported birthday announcements or age [53,80,90,108,110,112], (2) for tweets in which a user was wished a happy birthday [90], (3) for profiles that self-reported age [64,66,108,112], (4) for profiles that mentioned age-related keywords (eg, *grandparent*) [66,112], or (5) for manual annotation based on images or profile metadata [112,116,140] or (6) by subjectively perceiving age groups based on the content of individual tweets [73]. In 1 (5%) study [51], a mixture of self-reported information and demographic information of known individuals was used to label the data. Similar to studies on gender, the reported availability of the corpora was scarce. Only 5 (26%) studies reported that their data sets were available, 2 (40%) by request, 1 (20%) provided a link to the whole data set, and 2 (40%) provided a link to a sample of the corpus (Multimedia Appendix 6).

##### Features and Models

The studies used labeled age groups to evaluate classification models based on the features of the users’ profile metadata (eg, user names, bio, followers, and users followed) [51,53,64,80,112], a combination of their profile metadata and tweets (eg, n-grams, word embeddings, hashtags, and URLs) [73,83,90,94,95,108,110], tweet texts only [65,66,70,87], or images [80,108,112,116].

For automatic classification, most studies (12/19, 63%) used traditional supervised ML methods, including logistic regression [51,66,87,90,110], Bayesian probabilistic inference [64], random forests [80], bag of trees [70], or SVM [65,116], or a semisupervised approach, learning from label proportion [53]. Other studies (7/16, 37%) used deep learning methods such as convolutional neural networks [55,73,94,95], graph recursive neural networks [83], and multimodal deep learning networks [108,112]. The best-performing systems for each study are listed in Tables 4 and 5. Of the 19 studies, 1 (5%) [101] classified age based on a previously developed age lexicon and did not report any performance metrics.

**Table 4:** Top reported system performance for studies predicting the age of Twitter users using traditional machine learning (ML) methods. Result metrics are reflected in this table as reported in the original publications and are not directly comparable to each other. Reviews are ordered by the number of classification groups.

Study	Number of age groups	Age class detail (y)	Language	ML method	Reported performance	
					*F*_1_-score	Accuracy
Jurgens et al [80], 2017	N/A^a^	Continuous	English	RF^b^ regression	N/A	0.71
Volkova [110], 2017	2	18-23 and 25-30	English and Spanish	LogR^c^	N/A	0.77
Xiang et al [116], 2017	2	≤30 and >30	English	CPME^d^	N/A	0.74
Ardehaly and Culotta [53], 2018	2	<25 and >25	English	LLP^e^	N/A	0.78
Morgan-Lopez et al [90], 2017	3	13-17, 18-24, and >24	English	LogR	0.74	N/A
Arafat et al [51], 2020	3	≤24, 25-39, and ≥40	NR^f^	LogR	N/A	0.71
Cornelisse and Pillai [66], 2020	3	18-24, 25-54, and >55	English	LogR	0.78	N/A
Markov et al [87], 2017	5	18-24, 25-34, 35-49, 50-64, and >65	English, Spanish, Dutch, and Italian	LogR	N/A	0.56-0.65
Cheng et al [65], 2018	5	18-24, 25-34, 35-44, 45-54, and 55-64	English, Filipino, and Taglish	SVC^g^	0.61	0.86
Garcia-Guzman et al [70], 2020	4	18-24, 25-34, 35-49, and >50	English	Bag of trees	N/A	0.67
Chamberlain et al [64], 2017	10 (3 subgroups)	<12, 12-13, 14-15, 16-17, 18-24, 25-34, 35-44, 45-54, 55-64, and >64	English, Spanish, French, and Portuguese	Bayesian probability	0.31-0.86 (3 class)	N/A

^a^N/A: not applicable.

^b^RF: random forest.

^c^LogR: logistic regression.

^d^CPME: coupled projection matrix extraction.

^e^LLP: learning with label proportions.

^f^NR: not reported.

^g^SVC: support vector classifier.

**Table 5:** Top reported system performance for studies predicting the age of Twitter users using deep learning machine learning (ML) methods. Result metrics are reflected in this table as reported in the original publications and are not comparable to each other. Reviews are ordered by the number of classification groups.

Study	Number of age groups	Age class detail (y)	Language	ML method	Reported performance	
					*F*_1_-score	Accuracy
Guimaraes et al [73], 2017	2	13-19 and >20	English	CNN^a^	0.94	N/A^b^
Kim et al [83], 2017	2	Young (18-23) and old (25-30)	English	GRNN^c^	N/A	0.81
Vijayaraghavan et al [108], 2017	3	<30, 30-60, and >60	English	DMT^d^	0.82	N/A
Pandya et al [94], 2018	3	Dutch: <20, 20-40, and >40; English 1: 13-17, 18-40, and >40; and English 2: 13-17, 18-24, and >25	English and Dutch	CNN	0.61-0.87	N/A
Pandya et al [95], 2020	3	Dutch: <20, 20-40, and >40; English 1: 13-17, 18-40, and >40; and English 2: 13-17, 18-24, and >25	English and Dutch	CNN	0.82-0.87	N/A
Wang et al [112], 2019	4	≤18, 18-30, 30-40, and 40-99	Multilingual—28	mmDNN^e^	0.52	N/A
Bayot and Goncalves [55], 2017	5	18-24, 25-34, 35-49, 50-64, and ≥65	English and Spanish	CNN	N/A	0.43-0.55

^a^CNN: convolutional neural network.

^b^N/A: not applicable.

^c^GRNN: graph recurrent neural network.

^d^DMT: deep multimodal multitask.

^e^mmDNN: multimodal deep neural network.

##### Performance

Assessing the performance differences between studies using traditional ML methods and those using deep learning or neural networks is challenging owing to variations in classification criteria (eg, different age groupings and different number of classification categories) and the variety of performance metrics reported. However, for both methods, higher performance was noted when the problem was framed as a binary or ternary classification than as a larger multinomial classification.

### Studies Using Previously Developed Methods

#### Overview

Among the 74 included studies, there were 23 (31%) studies in which the detection of gender or age was secondary to their research, and previously developed methods were used to detect the demographic information of their cohort. Of the 23 studies, 13 (57%) used open-source models, and 10 (43%) used off-the-shelf software. More details about each study are given in the subsequent sections.

#### Open-Source Models

Of the 13 studies that used open-source models, 3 (4%) [74,99,100] drew upon an extant model [141] that used a predictive lexicon for the multiclass classification of age or gender for their applications. None of these studies created a validation corpus to assess the performance of the system, which was originally reported as 89.9% accuracy for gender and 0.84 Pearson correlation coefficient for age. One (1%) study [118] used the same text-based model [141] and an image model [142] to determine the age and gender of their cohort. When tested against their gold-standard corpus of self-reports from profile descriptions, they found that the imaging model performed best for gender (accuracy=90%-92%), whereas textual features gave the best results for age (accuracy=60%). A total of 3 (4%) studies [78,91,114] used demographer [115,139,143] for gender predictions, with 1 (33%) study [91] evaluating the performance against a set of users who had self-reported their gender in a survey, finding an *F*_1_-score of 0.869 for women and 0.770 for men. A total of 2 (3%) studies [61,62] used an ensemble classifier of previously developed models, with a reported accuracy of 0.83 and an *F*_1_-score of 0.83 [122]. Two (3%) other studies [67,120] used M3 [112] to detect gender and age, with 1 (50%) study validating the performance using a manually labeled data set, finding an accuracy of 95.9% and an *F*_1_-score of 0.957 for gender and an accuracy of 77.6% and an *F*_1_-score of 0.731 for age. One (1%) study [56] used Deep EXpectation of apparent age [144] for age and gender detection, which reported a validation error of 3.96 years for age and an 88% accuracy for gender. One (1%) study [98] used the rOPenSci *gender* package, and no assessment of performance was reported.

#### Off-the-Shelf Software

In the 10 studies that used off-the-shelf software, Face ++ was the most common software, being used in 6 (60%) studies [63,77,88,97,109,119]. The remaining studies used DemographicsPro [59,60], Microsoft Face API [84], and RapidMiner [103].

In 4 (40%) [88,97,103,109] of the 10 studies, no validation of performance was carried out, and a further 2 (20%) studies simply reported that DemographicsPro *requires* 95% confidence to make an estimation [59,60]. Other studies (n=4, 40%) compared with manual annotation and identified an accuracy of 82.8% for age using Face ++ [77], 68% for strict age groups, or 83% if the age groupings were relaxed [63]. The performance for age using Microsoft Face API was measured at 0.895 Gwet agreement coefficient (AC) [84], when compared with manually labeled data sets.

For gender, the studies (2/10, 20%) that measured performance against their own gold-standard labeled set of users recorded accuracies of 94.4% [77] or 88% [63] using Face ++. Other studies (3/10, 30%) [88,97,109] reported a confidence level of 95% +0.015 or –0.015 using Face ++ for gender prediction.

Only 1 (10%) [119] of the 10 studies went beyond manual annotation to create a gold standard and used multiple search techniques to manually verify age and gender, including LinkedIn profiles, electoral roll listings, personal websites, Twitter descriptions, and Twitter profile images. In this study, Face++ accuracy for age was reported as 40.4%, and Face++ accuracy for gender was reported as 44.8% (with a valid image accuracy of 32.5% for age and 87.7% for gender), and crowdsourcing annotation accuracy for age was 60.8% and for gender was 86.4% (with valid image accuracy of 56.1% for age and 93.9% for gender).

## Discussion

### Principal Findings

#### Overview

In this review, we aimed to provide an overview of recent ML methods used to predict the gender and age of Twitter users, as these are key demographics for epidemiology. Our review indicates that both tasks have been popular, but the identification of gender has received more attention than the identification of age. However, no de facto standards for research (ie, data collection and evaluation) have emerged, resulting in a large number of heterogeneous studies that are not directly comparable. Thus, it is not straightforward to conclude where the state-of-the-art stands for these tasks.

Our review found evidence of potential bias that impacts the quality and representativeness of the data used in the studies. One prevalent source of bias lies in the data collection and labeling processes. For instance, some studies may introduce systemic bias through the use of imprecise labeling methods such as name matching for labeling Twitter users’ gender. This approach can lead to mislabeling, especially for individuals with names that are culturally diverse or androgynous and introduce inaccuracies into the training data. Another problem is the introduction of sampling bias through the use of artificially balanced data sets, creating an unrepresentative sample of the Twitter population, which, in reality, has a skewed distribution, with certain age and gender groups being more prevalent than others.

It is important to address and limit these biases because when ML models are trained on biased data, they tend to replicate and amplify these biases in their predictions [145].

The prediction of demographic information is an important task to address to fully realize the potential advantages of using social media data, such as those of using Twitter data in health-related research. In the United States, the National Institute of Health has committed to including women participants in clinical studies and including sex as a biological variable, finding that the disaggregation of data by sex will allow for sex-based comparisons of results to identify any sex-based differences. A recent review [146] found that this disaggregation in the development of ML models led to the discovery of sex-based differences that improved the model performance for sex-specific cohorts. Age is also important, as it can correlate and be a factor in the course and progression of disease [146] or the effects of medication [147]. Given the significance of this information, accurate and reproducible models must be developed. One way to ensure the reproducibility of models is for researchers to make data and codes available, including annotation guidelines. In addition to model performance, studies that create annotated corpora should report annotator agreement measures to assess the quality of the corpora. Few of the included studies made their data or code available (Multimedia Appendices 3, 4, and 6).

A particular difficulty when comparing different systems comes from a lack of a *gold standard* that can be used to compare the systems. Some studies created their own corpora, collecting data randomly or based on keywords relevant to their studies. Others reused data sets from prior studies or shared tasks. Although outside the scope of this review, there have been shared tasks that aim to advance research through competition, focusing on gender and age prediction. A longstanding shared task focused on author profiling was hosted at the PAN workshop of CLEF [123-129]. More recently, Social Media Mining for Health (SMM4H), 2022, included 2 tasks for age detection, releasing new annotated corpora for the tasks [148]; several researchers reported using the corpora from these shared tasks. Testing and reporting performance metrics against these publicly available data sets, without alteration, would provide a comparable metric of different approaches. However, although reusing annotated corpora provides quick access to labeled data, it does have some limitations, including data loss over time as users delete their tweets, which not only reduces the size of the data but also can result in a data imbalance in the corpus.

**Figure 2:**
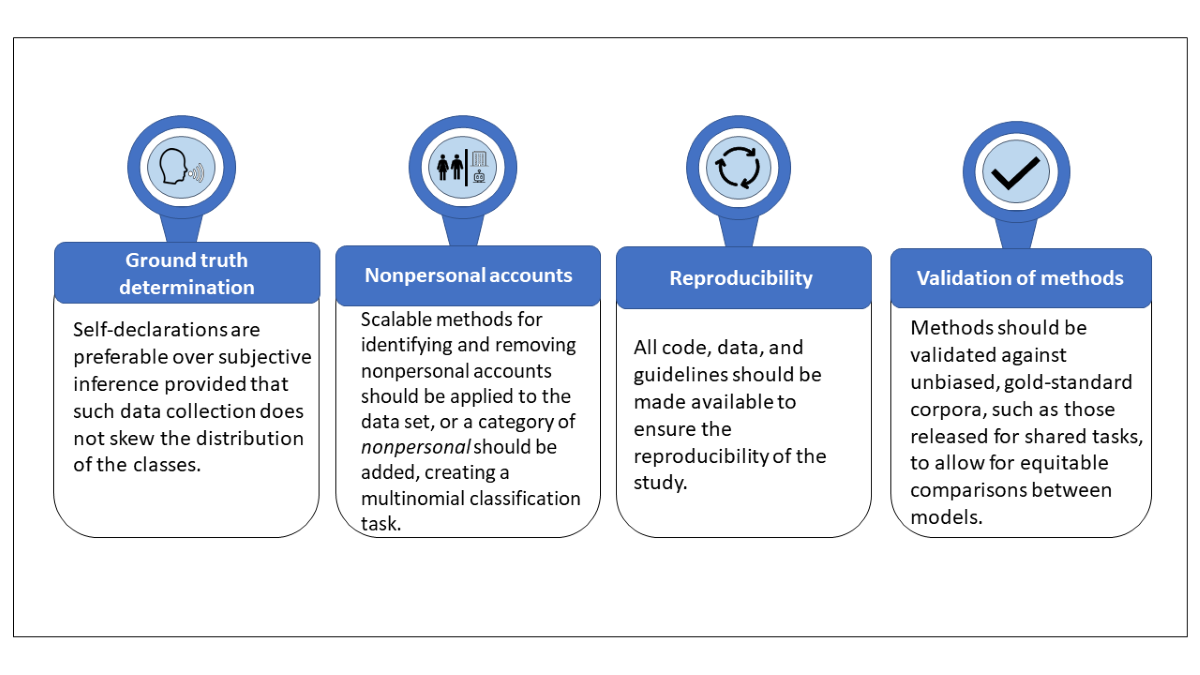
Summary of recommendations for best practices in the collection of training data, and the development and dissemination of age or gender machine learning prediction models.

A summary of our recommendations to reduce some potential bias in the data and improve the classification, reproducibility, and validation of the ML methods used can be found in Figure 2.

#### Gender Prediction

Almost all the included studies approached the gender prediction task as a binary classification task, identifying a user as either male or female. We note that even when focusing on binary gender classification, which is the prevalent approach, the task of gender prediction on Twitter could be better characterized as a multinomial classification task: given a user account, the classifier should return male, female, or *nonpersonal*. The last label (nonpersonal) can account for Twitter users representing organizations or bots. Although some studies attempted to identify and exclude nonpersonal accounts as a preprocessing step, other studies developed their systems using previously annotated data sets that were exclusively labeled as male or female users or removed nonpersonal accounts during annotation before training and testing. It is unknown how well these systems would perform when extended to unseen data that may contain nonpersonal accounts.

Excluding nonpersonal accounts, the ratio of male users to female users in the training data set is also important, as it should mimic the natural distribution of Twitter users, estimated to be 31.5% female users and 68.5% male users as of January 2021 [149]. However, some authors biased their collections using unconventional methods of collection or using artificially balanced data sets. The most conventional method to collect a set of Twitter accounts is to query for any tweet mentioning functional words without semantic meaning such as *of*, *the*, or *and* from the Twitter API. Whereas collecting Twitter users using functional or neutral keywords, a given language, or geographic areas resulted in a male:female ratio close to the ratio naturally observed on Twitter, other choices resulted in collections with different ratios. Such changes in ratios could have improved (or deteriorated) the training of the authors’ classifiers and biased their evaluations, which did not reflect the performance of their approach on a random sample of Twitter users.

All studies treated gender as a binary determination of male or female. Although some referenced the limitation of this approach, they opted to use these designations given the need to align their data with outside resources, such as the US census or social security administration data. We note that gender, unlike biological sex, is not necessarily binary as it is a social construct and has been shown to influence a person’s use of health care, interactions, therapeutic responses, disease perceptions, and decision-making [150]; this underlies the importance of expanding the efforts of classification beyond binary to improve accuracy and avoid misinterpreting results.

#### Age Prediction

The age prediction task generally had a lower performance than the gender prediction task. This was true for studies that developed their own models as well as those that used open-source or off-the-shelf software. This may be because most studies approached age prediction as a multiclass classification task. The proxies used, such as language, names, networks, or images, may have limited predictive value for age. In addition, the distribution of Twitter users means that any data set will be inherently imbalanced, providing few training examples for age groups at the tail end of the distribution. This data imbalance may lead to too few instances of the minority classes to effectively train the classifier. For classification models based on images, poor performance for age may be unsurprising given that it can be difficult for humans to discern age from a single image. In addition, photos may be subject to photo editing or enhancement or may not be a recent photograph of the user. Because of a lack of error analysis reports in the included studies, it is difficult to determine the source of the classification difficulty for age.

Performance aside, the fact that the number and range of age groups vary across studies suggests that a classification approach is not generalizable to all research applications. Identifying the exact age, rather than age groups, can generalize to applications that do not align with predefined groupings of binary or multiclass models; however, using high-precision rules to extract self-reports of exact age from the user’s profile metadata had been shown not to scale. As we worked on this study, we noted that none of the reviewed systems opted for extracting the exact age. To test the feasibility and utility of a generalizable system that extracts the exact age from a tweet in a user’s timeline using deep learning methods, separate from this study, our group developed a classification and extraction pipeline using the RoBERTa-Large model and a rule-based extraction model [151]. The system was trained and tested on 11,000 annotated tweets. The classification of tweets mentioning an age achieved an *F*_1_-score of 0.93, and the extraction of age from these tweets achieved an *F*_1_-score of 0.86. From a collection of 245,947 users, age was extracted for 54% using REPORTage. A shared task for the classification task ran at the SMM4H 2022 workshop, and we released the annotated data set. We did not include our approach in the scoping review, as there were no comparable systems published before the release of the exact age extraction approach as part of the SMM4H 2022 shared task.

#### Potential Bias of Differing Methods

The limitations of using names to distinguish between genders may promote bias, particularly if the names used for training do not represent the ethnic diversity of the population, and some cultures may have more unisex names than others, which cannot be used to distinguish genders. There can be a high degree of uncertainty for many users for whom gender cannot be classified by name; estimates by Sloan et al [152] are that 52% of users will be unclassified using this method. However, studies have suggested that the classifications made may be relatively accurate given that the data from UK Twitter demonstrates a high level of agreement with the UK census data [153]. Furthermore, when used alone, this heuristic may label some organization accounts, such as PAUL_BAKERY, as a person.

Relying on self-declarations may be prone to bias as well. For example, younger people are more likely to profess their age than older adults, as age may be more important to them. With respect to gender pronouns, these may be more likely to be declared by those in some occupations or age groups. Indeed, there may also be other biases to self-declarations of data based on culture, background, social class, or country of origin or residence.

Using users’ profile images for gender and age identification is challenging. Not all Twitter users provide a picture of themselves, with many opting for pictures of their pets, objects, children, scenery, or even celebrities. Identifying the gender and age of even those with pictures of themselves can be problematic if the quality of the pictures is poor, the pictures contain more than 1 face, or the pictures are not recent, particularly for predicting age. A comparison of systems using images to predict demographics [154] measured not only the accuracy in identifying age and gender but also the percentage of images in which a face could be detected, finding that only approximately 30% of Twitter users had a single detectable face.

Methods to filter out organizations in the studies included removing accounts with a large number of followers [71] or explicitly searching for organizations by matching username terms linked to economic activities, such as restaurant and hotel [49]. These methods remove accounts that do not represent a single user. However, they do not remove bots. Although one of the studies created a classifier to detect bots, the filtering of bots was limited to those identified in manual annotation, by simple heuristics, or nonexistent in many studies (Multimedia Appendix 5).

#### Validation of Age and Gender Proxies

For cases where age or gender are estimated, it is necessary to conduct validation exercises whereby the data are compared with a *gold-standard data set* to establish accuracy levels. For example, 1 study [119] that used off-the-shelf software also created a manually annotated gold-standard data set for measuring accuracy. This study found that although the accuracy of crowdsourcing was higher than that of software, the accuracy was only approximately 60% for age. This puts into question the use of manual annotations alone as a gold standard.

The most reliable way of generating a *gold standard* is to obtain the information directly from the user. This may be done in the form of direct correspondence with the user, such as messaging via social media or, the other way around, requesting Twitter handles in surveys that collect demographic data. Other methods for validation, such as manual extraction, may be less rigorous. However, these methods can be improved by multiple independent annotators, using experienced teams.

External validation of the model is also a vital step to assess how the model will perform on unseen data [155,156]. In a validation on a second data set, Yang et al [117] found that performance dropped in all but 2 of their models, stressing the importance of benchmarking existing systems on a targeted corpus. This step is equally important when using existing systems, so a range of expected performances can be reported and used in any analysis of the output.

In addition to the potential biases reported earlier, predicting the age and gender of Twitter users has some potential limitations that should be considered and, when possible, addressed to limit their effects. As evidenced by the performance results of the included studies, determining the precise age or age group of Twitter users solely based on their Twitter profiles and tweet content can be challenging. Although methods to extract a user’s self-reported age can be executed with high precision [151], predicting age, especially for more specific age groups, remains a complex task. Another limitation to consider is the potential for users to misrepresent their reported age or gender, which can introduce inaccuracies and affect the reliability of predictions based on user-supplied data. This phenomenon is not unique to Twitter and has been identified in other data sources such as surveys [157,158]. Many of the included studies used self-reported data to label their training data; therefore, any potential misrepresentations could be approached as a noisy label problem. There are numerous methods that can be used to manage the effect of label noise on classification models, such as distance learning or ensemble methods [159,160]. Furthermore, it is important to effectively address potential noise and uncertainty when using the output data for secondary analysis. Statistical techniques that can handle imprecise or uncertain data, such as Bayesian inference or fuzzy logic, can be valuable in this context. Using these methods, the analysis can better account for uncertain predictions, leading to more robust and reliable results. Finally, users’ age changes over time, and their profiles may not be updated accordingly, or the age tweet may be from an earlier year and not reflect their current age. Researchers should ensure that the users’ labeled age is contemporaneous with the other data included in the prediction model. Predicting the age and gender of Twitter users provides valuable insights, and most identified limitations presented by the data can be mitigated.

### Ethical Considerations

Several studies have shown that social media users generally do not have concerns about their data being used for research or even have favorable opinions about it [161,162]. However, the ethical frameworks for the use of these data are still being developed [163-165], and institutional review boards may deem the use of publicly available data, such as those collected from Twitter, as exempt from human participant research; however, it is incumbent on the researcher to consult with their institutional review boards or equivalent ethical committees to obtain such exemptions [165]. Although the data are publicly available, it is important to carefully consider potential ethical implications when predicting the age and gender of Twitter users. This process may raise privacy concerns, particularly when publishing data that may be considered sensitive, necessitating the protection of user identities and the anonymization of data to prevent reidentification [166]. Anonymizing the data may include removing user identifiers, modifying the tweet text, or generating synthetic tweets [165]. In addition, automated methods for predicting user age or gender have limitations and may result in misclassifications. Transparency regarding the limitations of the methods, algorithms, and data sources used in age and gender prediction are essential to report so that any use of these methods or data in secondary analysis can take such limitations into account. Although the prediction of age and gender may present some potential ethical concerns, it is important to recognize that there are also benefits to the use of these data for health research that can outweigh these concerns, such as eliciting insights into disease prevalence, patterns, and variations or distinguishing health behaviors and attitudes across different subgroups.

### Limitations

It is unlikely that we have identified all studies using off-the-shelf software, as we did not search for specific named software, but part of our remit was to identify the array of software used. We did not limit our inclusion to only studies that developed their own software; therefore, we have included studies that used proprietary software. These software products do not publish their methodologies; therefore, we are unable to directly compare these approaches with others.

We also included studies for which the prediction of age and gender was secondary to the primary focus of their study. These studies either used proprietary software, previously developed methods, or developed limited methods to predict demographic information. In general, these studies did not report the performance of their prediction methods on their data sets. Although some reported the original performance metrics of the methods used, it cannot be assumed that these methods will perform similarly across all data.

### Conclusions

The prediction of demographic data, such as age and gender, is an important step in increasing the value and application of social media data. Many methods have been reported in the literature with differing degrees of success. Although we sought to explore whether deep learning approaches would advance the performance for these tasks as they have been shown to do for other natural language processing tasks, many of the included studies used traditional ML methods. Although only explored by a handful of studies, deep learning methods appear to perform well for the prediction of a user’s gender or age. However, direct comparison of the published methods was impossible, as different test sets were used in the studies. This highlights the need for recently developed, publicly available gold-standard corpora, such as those released for shared tasks such as SMM4H or PAN-CLEF, to have unbiased data and baseline metrics to compare different approaches going forward.

## Appendix

Multimedia Appendix 1 PRISMA-ScR (Preferred Reporting Items for Systematic Reviews and Meta-Analyses Extension for Scoping Reviews) checklist.

Multimedia Appendix 2 Search strategies and results for individual databases.

Multimedia Appendix 3 Extracted data from the included studies predicting gender.

Multimedia Appendix 4 Extracted data from the included studies predicting age.

Multimedia Appendix 5 Information on the identification and removal of nonpersonal or bot accounts from the data set. Features used for annotation or prediction of gender or age.

Multimedia Appendix 6 Details of corpora created in the included studies and their reported availability.

## Abbreviations

API: application programming interface
CLEF: Conference and Labs of the Evaluation Forum
DNN: deep neural network
ML: machine learning
PICOS: Population, Intervention, Comparison, Outcomes, and Study Design
PRISMA-ScR: Preferred Reporting Items for Systematic Reviews and Meta-Analyses Extension for Scoping Reviews
SMM4H: Social Media Mining for Health
SVM: support vector machine

## Data Availability

The included studies are available on the web. The search strategy and extracted data on included studies are available in Multimedia Appendices 2-6.
