# Supplementary material for: Methods and Annotated Data Sets Used to Predict the Gender and Age of Twitter Users: Scoping Review
Source: J Med Internet Res. 2024 Mar 15;26:e47923. doi: 10.2196/47923 (PMC10980991; doi:10.2196/47923)
Supplement: Multimedia Appendix 2 [file jmir_v26i1e47923_app2.docx]

Table S2: Search strategies and results for individual databases.

| Database | Date Searched | Search Strategy | Results |
| --- | --- | --- | --- |
| ACL Anthology | 26/06/2020 | (Twitter OR tweet OR tweeting OR tweets OR retweet* OR tweep*) AND (gender OR age OR demograph*) | 5,080 records |
| ACM Digital Library | 26/06/2020 | [[Publication Title: twitter] OR [Publication Title: tweet] OR [Publication Title: tweeting] OR [Publication Title: tweets] OR [Publication Title: retweet*] OR [Publication Title: tweep*]] AND [[Publication Title: age] OR [Publication Title: gender] OR [Publication Title: demograph*] OR [Publication Title: minor*] OR [Publication Title: baby boomer*] OR [Publication Title: "generation x"] OR [Publication Title: "generation y"] OR [Publication Title: "generation z"] OR [Publication Title: "gen x"] OR [Publication Title: "gen y"] OR [Publication Title: “gen z$] OR [Publication Title: millennial*] OR [Publication Title: adult*] OR [Publication Title: teen*] OR [Publication Title: youth*] OR [Publication Title: adolescen*] OR [Publication Title: juvenile*] OR [Publication Title: young] OR [Publication Title: youngster*] OR [Publication Title: aged] OR [Publication Title: aging] OR [Publication Title: ageing] OR [Publication Title: elder*] OR [Publication Title: old] OR [Publication Title: retired] OR [Publication Title: older*] OR [Publication Title: geriatr*] OR [Publication Title: gerontolog*] OR [Publication Title: senior*] OR [Publication Title: senescen*] OR [Publication Title: retiree*] OR [Publication Title: sexagenarian*] OR [Publication Title: septuagenarian*] OR [Publication Title: octagenarian*] OR [Publication Title: nonagenarian*] OR [Publication Title: centenarian*] OR [Publication Title: supercentenarian*] OR [Publication Title: veteran*] OR [Publication Title: pensioner*] OR [Publication Title: male*] OR [Publication Title: female*] OR [Publication Title: men] OR [Publication Title: women]]  (Twitter OR tweet OR tweeting OR tweets OR retweet* OR tweep*) AND (age OR gender OR demograph* OR minor* OR baby boomer* OR "generation x" OR "generation y" OR "generation z" OR "gen x" OR "gen y" OR “gen z$ OR millennial* OR adult* OR teen* OR youth* OR adolescen* OR juvenile* OR young OR youngster* OR aged OR aging OR ageing OR elder* OR old OR retired OR older* OR geriatr* OR gerontolog* OR senior* OR senescen* OR retiree* OR sexagenarian* OR septuagenarian* OR octagenarian* OR nonagenarian* OR centenarian* OR supercentenarian* OR veteran* OR pensioner* OR male* OR female* OR men OR women) | 23 records |
| CINAHL Complete | 18/05/2021 | (Twitter OR tweet OR tweeting OR tweets OR retweet* OR tweep*) in Title  AND  (age OR gender OR demograph* OR minor* OR baby boomer* OR generation X OR generation Y OR generation Z OR gen X OR gen Y OR gen Z OR millennial* OR adult* OR teen* OR youth* OR adolescen* OR juvenile* OR young OR youngster* OR aged OR aging OR ageing OR elder* OR old OR retired OR older* OR geriatr* OR gerontolog* OR senior* OR senescen* OR retiree* OR sexagenarian* OR septuagenarian* OR octagenarian* OR nonagenarian* OR centenarian* OR supercentenarian* OR veteran* OR pensioner* OR male* OR female* OR men OR women OR sex) in Title | 65 records |
| Embase <1980 to 2021 Week 19> | 18/05/2021 | 1  (Twitter or tweet or tweeting or tweets or retweet* or tweep*).ti,ab. (5718) 2  demography/ (279727) 3  demograph*.ti,ab. (586081) 4  age distribution/ (147433) 5  exp groups by age/ (11355955) 6  "minor (person)"/ (706) 7  (minor* or baby boomer* or generation X or generation Y or generation Z or gen X or gen Y or gen Z or millennial* or adult*).ti,ab. (2068747) 8  (teen* or youth* or adolescen* or juvenile* or (young adj2 (adult* or person* or individual* or people* or population* or man or men or wom#n)) or youngster*).ti,ab. (749837) 9  (aged or aging or ageing or elder* or old or retired or older* or geriatr* or gerontolog* or senior* or senescen* or retiree* or sexagenarian* or septuagenarian* or octagenarian* or nonagenarian* or centenarian* or supercentenarian* or veteran* or pensioner*).ti,ab. (3205034) 10  age.ti,ab. (3738110) 11  sex ratio/ (73055) 12  exp "gender and sex"/ (1024490) 13  gender.ti,ab. (545242) 14  (male* and female*).ti,ab. (747966) 15  (men and women).ti,ab. (412683) 16  sex.ti,ab. (733711) 17  or/2-16 (14116212) 18  1 and 17 (2396) 19  exp algorithm/ (430675) 20  exp machine learning/ (249869) 21  exp algorithm/ (430675) 22  Neural Network*.ti,ab. (71079) 23  deep learning.ti,ab. (18237) 24  back propagation.ti,ab. (2883) 25  regression tree.ti,ab. (3502) 26  class prior.ti,ab. (103) 27  Conditional Random Field*.ti,ab. (632) 28  Decision Table.ti,ab. (137) 29  discriminating.ti,ab. (38906) 30  Decision Stump.ti,ab. (16) 31  Elastic Net.ti,ab. (1774) 32  Factor Graph Model*.ti,ab. (7) 33  Gaussian Mixture Model*.ti,ab. (1461) 34  Gaussian Process.ti,ab. (1315) 35  Higher Order Singular Value Decomposition.ti,ab. (53) 36  Instance-based Learning.ti,ab. (45) 37  J48.ti,ab. (295) 38  JRip.ti,ab. (29) 39  machine learning.ti,ab. (46762) 40  algorithms.ti,ab. (129440) 41  automati*.ti,ab. (168866) 42  Multi-task Learning.ti,ab. (340) 43  Non-negative Matrix Factorization.ti,ab. (1039) 44  Non-negative Tensor Factorization.ti,ab. (18) 45  Perceptron.ti,ab. (3042) 46  Random Sample Consensus.ti,ab. (141) 47  Rule Based.ti,ab. (4399) 48  Radial Basis Function Network.ti,ab. (210) 49  learning machine.ti,ab. (1426) 50  Reptree.ti,ab. (30) 51  Skip Gram.ti,ab. (48) 52  Support Vector Machine.ti,ab. (16434) 53  XGBoost.ti,ab. (861) 54  Stochastic gradient descent.ti,ab. (340) 55  Artificial intelligence.ti,ab. (14435) 56  Language Processing.ti,ab. (8475) 57  bag of words.ti,ab. (353) 58  text mining.ti,ab. (2787) 59  gated recurrent unit.ti,ab. (165) 60  word2vec.ti,ab. (170) 61  Determin*.ti. (351544) 62  Infer*.ti. (74214) 63  Ascertain.ti. (294) 64  Establish*.ti. (55917) 65  Predict*.ti. (507527) 66  Classify.ti,ab. (63529) 67  Classification.ti,ab. (453687) 68  Classifier.ti,ab. (27837) 69  intrinsic bias.ti,ab. (103) 70  facial recognition.ti,ab. (985) 71  extract*.ti. (182221) 72  identify.ti. (34537) 73  identifying.ti. (36813) 74  identified.ti. (28390) 75  or/20-74 (2373702) 76  18 and 75 (281) 77  twitter user*.ti,ab. (397) 78  75 and 77 (113) 79  76 or 78 (358) 80  remove duplicates from 79 (353) | 353 records |
| Google Scholar | 26/06/2020 | twitter age gender demographic | 767,000. Sifted until 100 non-relevant records |
| [IEEE/IET Electronic Library (IEEE Xplore)](http://libproxy.york.ac.uk/login?url=https://ieeexplore.ieee.org/Xplore/home.jsp) | 26/06/2020 | (Twitter OR tweet OR tweeting OR tweets OR retweet* OR tweep*) in Title  AND  (age OR gender OR demograph*) in Title | 25 records |
| LISTA | 18/05/2021 | (Twitter OR tweet OR tweeting OR tweets OR retweet* OR tweep*) in Title  AND  (age OR gender OR demograph* OR minor* OR baby boomer* OR generation X OR generation Y OR generation Z OR gen X OR gen Y OR gen Z OR millennial* OR adult* OR teen* OR youth* OR adolescen* OR juvenile* OR young OR youngster* OR aged OR aging OR ageing OR elder* OR old OR retired OR older* OR geriatr* OR gerontolog* OR senior* OR senescen* OR retiree* OR sexagenarian* OR septuagenarian* OR octagenarian* OR nonagenarian* OR centenarian* OR supercentenarian* OR veteran* OR pensioner* OR male* OR female* OR men OR women OR sex) in Title | 55 records |
| Proquest Dissertations & Theses: UK & Ireland | 18/05/2021 | (Twitter OR tweet OR tweeting OR tweets OR retweet* OR tweep*) in Document title  AND  (age OR gender OR demograph* OR minor* OR baby boomer* OR generation X OR generation Y OR generation Z OR gen X OR gen Y OR gen Z OR millennial* OR adult* OR teen* OR youth* OR adolescen* OR juvenile* OR young OR youngster* OR aged OR aging OR ageing OR elder* OR old OR retired OR older* OR geriatr* OR gerontolog* OR senior* OR senescen* OR retiree* OR sexagenarian* OR septuagenarian* OR octagenarian* OR nonagenarian* OR centenarian* OR supercentenarian* OR veteran* OR pensioner* OR male* OR female* OR men OR women OR sex) in Document title | 69 records |
| Ovid MEDLINE(R) and Epub Ahead of Print, In-Process & Other Non-Indexed Citations and Daily <1946 to May 17, 2021> | 18/05/2021 | 1  (Twitter or tweet or tweeting or tweets or retweet* or tweep*).ti,ab. (4310) 2  demography/ (61705) 3  demograph*.ti,ab. (352939) 4  age distribution/ (67431) 5  exp age groups/ (9394176) 6  minors/ (2642) 7  (minor* or baby boomer* or generation X or generation Y or generation Z or gen X or gen Y or gen Z or millennial* or adult*).ti,ab. (1619423) 8  (teen* or youth* or adolescen* or juvenile* or (young adj2 (adult* or person* or individual* or people* or population* or man or men or wom#n)) or youngster*).ti,ab. (602179) 9  (aged or aging or ageing or elder* or old or retired or older* or geriatr* or gerontolog* or senior* or senescen* or retiree* or sexagenarian* or septuagenarian* or octagenarian* or nonagenarian* or centenarian* or supercentenarian* or veteran* or pensioner*).ti,ab. (2373166) 10  age.ti,ab. (2403830) 11  exp sex distribution/ (65276) 12  exp Gender Identity/ (20455) 13  gender.ti,ab. (338094) 14  (male* and female*).ti,ab. (502290) 15  (men and women).ti,ab. (301108) 16  sex.ti,ab. (546438) 17  or/2-16 (11685101) 18  1 and 17 (1122) 19  classification/ (10421) 20  exp algorithms/ (347247) 21  Data Mining/ (9160) 22  Neural Network*.ti,ab. (58468) 23  deep learning.ti,ab. (15501) 24  back propagation.ti,ab. (2303) 25  regression tree.ti,ab. (2638) 26  class prior.ti,ab. (50) 27  Conditional Random Field*.ti,ab. (661) 28  Decision Table.ti,ab. (90) 29  discriminating.ti,ab. (31208) 30  Decision Stump.ti,ab. (10) 31  Elastic Net.ti,ab. (1181) 32  Factor Graph Model*.ti,ab. (7) 33  Gaussian Mixture Model*.ti,ab. (1248) 34  Gaussian Process.ti,ab. (1324) 35  Higher Order Singular Value Decomposition.ti,ab. (47) 36  Instance-based Learning.ti,ab. (43) 37  J48.ti,ab. (200) 38  JRip.ti,ab. (23) 39  machine learning.ti,ab. (38527) 40  algorithms.ti,ab. (104671) 41  automati*.ti,ab. (132916) 42  Multi-task Learning.ti,ab. (309) 43  Non-negative Matrix Factorization.ti,ab. (789) 44  Non-negative Tensor Factorization.ti,ab. (16) 45  Perceptron.ti,ab. (2613) 46  Random Sample Consensus.ti,ab. (117) 47  Rule Based.ti,ab. (3895) 48  Radial Basis Function Network.ti,ab. (175) 49  learning machine.ti,ab. (912) 50  Reptree.ti,ab. (27) 51  Skip Gram.ti,ab. (50) 52  Support Vector Machine.ti,ab. (13313) 53  XGBoost.ti,ab. (689) 54  Stochastic gradient descent.ti,ab. (260) 55  Artificial intelligence.ti,ab. (11535) 56  Language Processing.ti,ab. (7111) 57  bag of words.ti,ab. (324) 58  text mining.ti,ab. (2569) 59  gated recurrent unit.ti,ab. (140) 60  word2vec.ti,ab. (160) 61  Determin*.ti. (364254) 62  Infer*.ti. (66596) 63  Ascertain.ti. (264) 64  Establish*.ti. (47929) 65  Predict*.ti. (357533) 66  Classify.ti,ab. (47150) 67  Classification.ti,ab. (340482) 68  Classifier.ti,ab. (20726) 69  intrinsic bias.ti,ab. (86) 70  facial recognition.ti,ab. (755) 71  extract*.ti. (155990) 72  identify.ti. (24499) 73  identifying.ti. (28622) 74  identified.ti. (23424) 75  or/19-74 (1900871) 76  18 and 75 (175) 77  twitter user*.ti,ab. (353) 78  75 and 77 (109) 79  76 or 78 (253) | 253 records |
| APA PsycInfo <1987 to May Week 2 2021> | 18/05/2021 | 1  (Twitter or tweet or tweeting or tweets or retweet* or tweep*).ti,ab. (3362) 2  demograph*.ti,ab. (122659) 3  (minor* or baby boomer* or generation X or generation Y or generation Z or gen X or gen Y or gen Z or millennial* or adult*).ti,ab. (476915) 4  (teen* or youth* or adolescen* or juvenile* or (young adj2 (adult* or person* or individual* or people* or population* or man or men or wom#n)) or youngster*).ti,ab. (358974) 5  (aged or aging or ageing or elder* or old or retired or older* or geriatr* or gerontolog* or senior* or senescen* or retiree* or sexagenarian* or septuagenarian* or octagenarian* or nonagenarian* or centenarian* or supercentenarian* or veteran* or pensioner*).ti,ab. (590117) 6  age.ti,ab. (480959) 7  gender.ti,ab. (211626) 8  (male* and female*).ti,ab. (157447) 9  (men and women).ti,ab. (96265) 10  sex.ti,ab. (143406) 11  or/2-10 (1552274) 12  1 and 11 (828) 13  Neural Network*.ti,ab. (16828) 14  deep learning.ti,ab. (1500) 15  back propagation.ti,ab. (476) 16  regression tree.ti,ab. (330) 17  class prior.ti,ab. (20) 18  Conditional Random Field*.ti,ab. (84) 19  Decision Table.ti,ab. (39) 20  discriminating.ti,ab. (6938) 21  Decision Stump.ti,ab. (0) 22  Elastic Net.ti,ab. (175) 23  Factor Graph Model*.ti,ab. (1) 24  Gaussian Mixture Model*.ti,ab. (255) 25  Gaussian Process.ti,ab. (256) 26  Higher Order Singular Value Decomposition.ti,ab. (4) 27  Instance-based Learning.ti,ab. (64) 28  J48.ti,ab. (26) 29  JRip.ti,ab. (7) 30  machine learning.ti,ab. (6235) 31  algorithms.ti,ab. (14019) 32  automati*.ti,ab. (33432) 33  Multi-task Learning.ti,ab. (76) 34  Non-negative Matrix Factorization.ti,ab. (149) 35  Non-negative Tensor Factorization.ti,ab. (3) 36  Perceptron.ti,ab. (509) 37  Random Sample Consensus.ti,ab. (7) 38  Rule Based.ti,ab. (1894) 39  Radial Basis Function Network.ti,ab. (35) 40  learning machine.ti,ab. (473) 41  Reptree.ti,ab. (3) 42  Skip Gram.ti,ab. (20) 43  Support Vector Machine.ti,ab. (1786) 44  XGBoost.ti,ab. (26) 45  Stochastic gradient descent.ti,ab. (57) 46  Artificial intelligence.ti,ab. (3855) 47  Language Processing.ti,ab. (5575) 48  bag of words.ti,ab. (103) 49  text mining.ti,ab. (548) 50  gated recurrent unit.ti,ab. (16) 51  word2vec.ti,ab. (28) 52  Determin*.ti. (21937) 53  Infer*.ti. (9275) 54  Ascertain.ti. (32) 55  Establish*.ti. (4278) 56  Predict*.ti. (78877) 57  Classify.ti,ab. (9485) 58  Classification.ti,ab. (52361) 59  Classifier.ti,ab. (3470) 60  intrinsic bias.ti,ab. (20) 61  facial recognition.ti,ab. (658) 62  extract*.ti. (2363) 63  identify.ti. (2814) 64  identifying.ti. (7663) 65  identified.ti. (2351) 66  or/13-65 (257359) 67  12 and 66 (85) 68  twitter users.ti,ab. (260) 69  66 and 68 (43) 70  67 or 69 (115) | 115 records |
| Science Citation Index (SCI), Social Science Citation Index (SSCI), Conference Proceedings Citation Index – Science, Conference Proceedings Citation Index – Social Science and Humanities, Emerging Sources Citation Index (ESCI) --2015-present | 18/05/2021 | (TI=(determin* OR infer* OR ascertain OR establish* OR predict* OR extract* OR identify OR identifying OR identified) OR TS=(classify OR classification OR Classifier OR intrinsic bias OR facial recognition OR algorithm* OR machine learning OR Neural Network* OR deep learning OR back propagation OR regression tree OR class prior OR Conditional Random Field* OR Decision Table OR discriminating OR Decision Stump OR Elastic Net OR Factor Graph Model* OR Gaussian Mixture Model* OR Gaussian Process OR Higher Order Singular Value Decomposition OR Instance-based Learning OR J48 OR JRip OR automati* OR Multi-task Learning OR Non-negative Matrix Factorization OR Non-negative Tensor Factorization OR Perceptron OR Random Sample Consensus OR Rule Based OR Radial Basis Function Network OR learning machine OR Reptree OR Skip Gram OR Support Vector Machine OR XGBoost OR Stochastic gradient descent OR Artificial intelligence OR Language Processing OR bag of words OR text mining OR gated recurrent unit OR word2vec)) AND TS=(Twitter OR tweet OR tweeting OR tweets OR retweet* OR tweep*) AND TI=(age OR gender OR demograph* OR minor* OR baby boomer* OR generation X OR generation Y OR generation Z OR gen X OR gen Y OR gen Z OR millennial* OR adult* OR teen* OR youth* OR adolescen* OR juvenile* OR young OR youngster* OR aged OR aging OR ageing OR elder* OR old OR retired OR older* OR geriatr* OR gerontolog* OR senior* OR senescen* OR retiree* OR sexagenarian* OR septuagenarian* OR octagenarian* OR nonagenarian* OR centenarian* OR supercentenarian* OR veteran* OR pensioner* OR male* OR female* OR men OR women OR sex) | 197 records |
| Zetoc | 26/06/2020 | Multiple searches of the title field were carried out:  Twitter AND gender (16 hits)  Twitter AND age (25 hits)  Twitter AND Demographics (5 hits)  Twitter AND Demographic (15 hits) | 61 records hits (including duplicates) |
